# Effect of intrauterine granulocyte-colony stimulating factor administration on in vitro fertilization outcome in women with moderate-to-severe endometriosis: An RCT

**DOI:** 10.18502/ijrm.v19i8.9621

**Published:** 2021-09-09

**Authors:** Ladan Kashani, Ashraf Moini, Tayebeh Esfidani, Nazila Yamini, Shima Mohiti

**Affiliations:** Department of Obstetrics and Gynecology, Arash Womens’ Hospital, Tehran University of Medical Sciences, Tehran, Iran.

**Keywords:** G-CSF, In vitro fertilization, Endometriosis, Pregnancy.

## Abstract

**Background:**

Nearly 25-50% of infertile women have endometriosis. There are reports of disorders in the expression of granulocyte colony-stimulating factor (G-CSF) receptors in women with endometriosis.

**Objective:**

To examine the effect of intrauterine administration of G-CSF in in vitro fertilization (IVF) cycles on the fertility rate of infertile women with moderate-to-severe endometriosis.

**Materials and Methods:**

This clinical trial was conducted on 66 infertile women with moderate-to-severe endometriosis, undergoing IVF and intracytoplasmic sperm injection (ICSI). The participants were allocated into two groups via simple randomization: the G-CSF (n = 27) and control (n = 39) groups. In the G-CSF intervention group, on the oocyte pick-up day, immediately after an ovarian puncture, 300 μg of G-CSF was injected through a transcervical catheter under abdominal ultrasound guide to visualize flushing into the uterine cavity. Women in the control group received no intervention. The two groups were evaluated for clinical pregnancy.

**Results:**

No significant difference was noted in the demographic characteristics of the two groups. The rate of clinical pregnancy was 28.2% in the control group and 25.9% in the G-CSF group, indicating no significant difference (p = 0.83).

**Conclusion:**

The results showed that the intrauterine injection of G-CSF had no effects on pregnancy in women with stage-3/4 endometriosis undergoing IVF.

## 1. Introduction

Endometriosis is defined as a disorder in which endometrial tissue is present outside the uterine cavity. This disorder occurs in about 5-10% of the female population and 25-50% of infertile women (1). However, the etiology of endometriosis is not yet clear (2). Strong biological evidence suggests differences in the stem-cell content, hormone sensitivity, cell adhesion, cell invasion, cellular proliferation, and angiogenesis in the endometrium of women with endometriosis compared with healthy individuals. The endometrium of women with endometriosis is resistant to the selective activities of progesterone, affecting decidualization and modulation of local inflammation during implantation (3).

Granulocyte colony-stimulating factor (G-CSF) is a glycoprotein that mainly stimulates the production of granulocytes. It is a polypeptide amino acid that may affect endometrial decidualization, differentiation of stem cells, and trophoblast migration and formation. Also, it may facilitate endometrial reconstruction by improving angiogenesis and decreasing cellular death. This glycoprotein may play an important role in implantation and maintenance of pregnancy through temporary immune response suppression by affecting lymphocytes, macrophages, and type-II T-helper cells (4).

Studies have mostly focused on the role of G-CSF in repeated implantation failure and low endometrial thickness despite treatment in infertile women undergoing in vitro fertilization (IVF) cycles (5-7). There are reports of disorders in the expression of G-CSF receptors in women with endometriosis (5). To the best of our knowledge, no clinical trial has yet investigated the effect of G-CSF on infertile women with endometriosis. Overall, a good-quality embryo, a receptive endometrium at the time of implantation, and an appropriate method of embryo transfer determine the success of assisted reproductive technology (ART) cycles. Considering the high prevalence of endometriosis in infertile women and the possible effects of this disorder on endometrial receptivity, this study was conducted to examine the effect of intrauterine administration of G-CSF in IVF cycles on the fertility rate of infertile women with moderate-to-severe endometriosis.

## 2. Materials and Methods

### Design 

This single-center randomized controlled clinical trial was conducted at the Arash Women's Hospital, Tehran, Iran between January 2019 and September 2019.

### Study population

This study included a total of 66 infertile women with endometriosis, who underwent IVF/ICSI for the first time (intracytoplasmic sperm injection) and met the inclusion and exclusion criteria of the study. The inclusion criteria were infertility due to endometriosis; the first time IVF aged 18-40 yr; and nulliparous with moderate-to-severe endometriosis. Endometriosis was diagnosed via laparoscopy or transvaginal ultrasound (TVUS; Affiniti 70 w, Philips) in the past six months. Moderate-to-severe endometriosis was characterized by endometrial glands and stroma at least 5 mm beneath the peritoneum or an ovarian endometriotic cyst (endometrioma) (8).

The exclusion criteria were: (1) male infertility; (2) evidence of a significant decrease in the ovarian reserve (follicle-stimulating hormone [FSH] > 11, anti-Müllerian hormone < 0.5, and decreased antral follicle count < 4-6 on day three of the menstrual cycle); (3) endocrine disorders (i.e., diabetes, thyroid diseases, hyperprolactinemia, and hypothalamic amenorrhea); (4) confirmed clinical or immunological diseases (i.e., systemic lupus erythematosus, rheumatoid arthritis, antiphospholipid syndrome, and cardiovascular, hepatic, or renal diseases); and (5) congenital uterine anomalies or uterine cavity disorders (i.e., bicornuate uterus, unicornuate uterus, Asherman syndrome, myoma, and polyps). Candidate donors (i.e., ovum donation and surrogate uterus) were also excluded from the study. To detect a 35% increase in the rate of clinical pregnancy in the G-CSF group, a sample size of 33 participants per group was necessary at a two-sided 5% significance level and a power of 80%.

### Random allocation, concealment, and blinding

After the doctor declared the eligibility of patients, the type of intervention was determined by the gynecologist assistant using simple randomization, dividing the patients into either the G-CSF or no-intervention groups. In this method, the RANDBETWEEN function in the Excel program was used to generate a random number between one and two. This was repeated 66 times. Blinding for patients was not possible because the patients were aware of the type of intervention. Evaluation of the final results was done by a physician who was blind to the intervention type. A statistician who was blind to the allocation analyzed the results.

### Intervention

The long gonadotropin-releasing hormone (GnRH) agonist protocol was used for ovulation stimulation in all participants. The ovulation stimulation cycle started from the luteal phase before the stimulation cycle. On the second day of the cycle, ovarian stimulation with GnRH was initiated. The monitoring cycle started five-seven days after the ovarian stimulation, and TVUS was performed every two-four days to adjust the gonadotropin dosage. In each monitoring cycle, the number and size of follicles were recorded. When at least two 17-mm follicles were visualized, ovulation was triggered using 10,000 IU of human chorionic gonadotropin (hCG). Next, oocyte pick-up and retrieval of follicles > 10 mm were carried out. On the oocyte pick-up day, immediately after an ovarian puncture, 300 μg of G-CSF (PDgrastimⓇ, Iran) in a prefilled syringe containing 300 μg of filgrastim in 0.5 mL solution (Pooyesh Darou, Iran) was injected through a transcervical catheter under abdominal ultrasound guide to visualize flushing into the uterine cavity and ensure that the catheter did not pass the internal os. Injections were performed by a single person. Subjects in the control group received no intervention.

The participants were followed-up for abdominal pain and fever after 48 hr. Fresh embryo transfer was carried out after three days during the same cycle. The number of transferred embryos was determined based on maternal age and quality of the embryos; one-three embryos were transferred accordingly. If the participants had vaginal bleeding or spotting, low endometrial thickness, or irregular endometrial polyps, the ART cycle was canceled and embryo transfer was not carried out. The participants were then evaluated for biochemical and clinical pregnancies. While a biochemical pregnancy was defined as a pregnancy detected by measuring the serum beta-hCG, a clinical pregnancy was confirmed by ultrasonographic observation of one or more gestational sacs (8) 21 days after embryo transfer.

### Ethical considerations

The study protocol was approved by the Ethics Committee of Tehran University of Medical Sciences (Code: IR.TUMS.MEDICINE.REC.1397.787). Informed consent was obtained from all participants.

### Statistical analysis

Data analysis was performed using SPSS version 18.0 (SPSS Inc., Chicago, Ill, USA). Qualitative data are presented as frequency and number (%) while qualitative data are reported as mean and standard deviation (SD). Numerical variables were compared using student's *t* test. Also, Chi-square (χ${}^{2}$) test was used for comparing the categorical data. P-value < 0.05 were considered statistically significant.

## 3. Results

**Figure 1:**
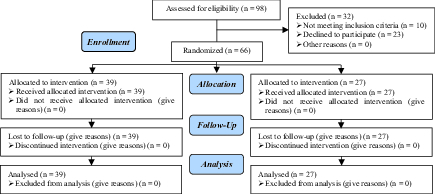
Flow diagram of participants.

Data of 39 women in the control group and 27 women in the G-CSF group were compared in this study (Figure 1). Endometriosis was diagnosed by vaginal ultrasound in 19 (79.2%) and 30 (90.9%) women in the G-CSF and control groups, respectively. There was no significant difference in the method of endometriosis diagnosis between the two groups (p = 0.2). Participants in both groups were comparable in terms of age, body mass index (BMI), day-three FSH level, number of transferred embryos, and number of retrieved oocytes (p > 0.05) (Table I).

The IVF cycle was canceled in nine (23.7%) women in the control group and seven (25.9%) in the G-CSF group, indicating no significant difference in the number of canceled cycles between the two groups. The cause of IVF cancelation was low endometrial thickness (n = 6), endometrial polyps (n = 2), and lack of cleavage (n = 1) in the control group and irregular endometrium (n = 2), lack of cleavage (n = 2), low endometrial thickness (n = 1), and spotting (n = 2) in the G-CSF group.

The rates of chemical (p = 0.93) and clinical (p = 0.83) pregnancies were not significantly different between the two groups (Table II). Also, after excluding women with canceled IVF due to cysts (based on the per-protocol analysis), the rate of clinical pregnancy was not significantly different between the two groups (control group: n = 11, 33.3%; G-CSF group: n = 7, 29.2%; p = 0.73).

**Table 1:** Baseline demographic profile and IVF characteristics of the two groups of the study


	**Control group (n = 39)**	**G-CSF group (n = 27)**	**P-value**
**Age (yr)**	30.92 ± 5.4	31.34 ± 3.2	0.68*
**BMI (Kg/m${}^{2}$)**	26.57 ± 4.03	25.20 ± 4.03	0.24*
**FSH (day 3)**	6.31 ± 1.6	6.83 ± 3.5	0.45*
**Number of oocytes retrieved**	6.66 ± 4.8	7.96 ± 4.12	0.26*
**Number of embryo transferred**	1.58 ± 1.01	1.70 ± 1.01	0.65*
**Cycle cancellation **	9 (23.1)	6 (22.2)	0.93${}^{\#}$
*Data presented as Mean ± SD, Student's *t* test, ${}^{\#}$Data presented as number (%), Chi-square test, IVF: In vitro fertilization, BMI: Body mass index, FSH: Follicle-stimulating hormone, G-CSF: Granulocyte-colony stimulating factor			

**Table 2:** Primary outcomes


****	**Control group (n = 39)**	**G-CSF group (n = 27)**	**P-value**
**Biochemical pregnancy **	12 (36.4)	9 (37.5)	0.93*
**Clinical pregnancy**	11 (28.9)	7 (25.9)	0.78*
*Data presented as number (%), Chi-square test, G-CSF: Granulocyte-colony stimulating factor			

## 4. Discussion

This study showed that the intrauterine injection of G-CSF was not associated with the clinical pregnancy rate on women with stage-3/4 endometriosis undergoing IVF. Generally, G-CSF is a recently discovered cytokine. It was first identified in rats in 1983, and its human form (hG-CSF) was cloned in 1986 (9, 10). G-CSF receptors are found in different types of non-hematopoietic cells, such as endothelial cells, placental cells, trophoblast cells, and luteinized granulosa cells (11). G-CSF receptors are expressed on the surface of trophoblast cells and luteinized granulosa cells (12). It is known that colony stimulating factor can regulate endometrial growth. The macrophage colony-stimulating factor is involved in endometrial development and affects the proliferation of endometrial epithelial cells (13). However, the mechanism of action of G-CSF must be clarified in clinical studies of G-CSF in reproductive medicine (14).

A review of the literature revealed that no study has yet examined the effectiveness of intrauterine G-CSF injection in women with endometriosis receiving IVF. In a study in 2011, a new option for the treatment of thin endometrium was introduced. Intrauterine injection of G-CSF increased the endometrial thickness in four IVF patients who did not respond to conventional treatments, and all of these women became pregnant (15). Other studies failed to find significant effects in women with a thin endometrium (16, 17).

Conversely, some studies have shown that routine use of G-CSF for women with a normal endometrium undergoing IVF does not have a positive effect on the IVF outcomes (6, 18). In the present study, intrauterine administration of G-CSF was performed in women with endometriosis. We did not observe any significant differences between the two groups in terms of chemical or clinical pregnancy rates. Many studies have reported lower pregnancy rates in women with endometriosis compared to healthy controls (19). An inverse correlation has been found between the success rate of IVF and stage-3/4 endometriosis (20). Moreover, IVF studies have shown that women with advanced endometriosis have a poorer ovarian reserve, lower-quality embryos and oocytes, and weaker implantation (21). In the present study, we selected infertile women with moderate-to-severe endometriosis.

There is currently little information about the mechanisms of infertility in women with endometriosis. The suggested mechanisms include altered folliculogenesis, ovarian disorders, low-quality oocytes, luteal-phase defects, abnormal embryogenesis, and endometrial receptivity disorders (19). Inadequate knowledge of the normal physiological mechanisms of implantation makes it difficult to determine why women with endometriosis may have a lower implantation capacity, resulting in decreased fertility (22). The mechanisms suggested for implantation dysfunction include changes in the expression of integrins and interleukins (23). Also, disorders in the regulation of other selective genes in the endometrium of women with endometriosis may result in embryo implantation disorders, embryo toxicity, immune disorders, and apoptosis during the implantation window. Other suggested mechanisms include dysfunctions in the progesterone zone and receptors and imbalance in the level of different cytokines, interleukins, and growth factors (19).

TVUS is highly useful for the detection of deep infiltrating endometriosis before surgery (24). In our clinic, TVUS was performed by an experienced infertility fellow. Therefore, the sensitivity and specificity of TVUS for the diagnosis of endometriosis increased. In line with our study, Pop-Trajkovic and colleagues showed that the cancellation rate of IVF cycles in women with advanced endometriosis was higher than women with stage-1/2 endometriosis or tubal-factor infertility (25), which is probably due to the endometriosis itself. It should be noted that, because the sample size of the present study was small, there might have been some random errors, and the lack of an observed relationship could have been a random finding. Also, the presence of unknown confounders may explain the lack of an observed relationship.

## 5. Conclusion

In conclusion, G-CSF may have no significant effect on endometrial function in women with endometriosis. The pregnancy rate is generally affected by factors, such as maternal age, number of embryos, and number of high-quality embryos transferred, which were similar in the two groups. Overall, the design of this study (a single-blind randomized clinical trial) is its major strength. This study also had some limitations, such as the small sample size and the lack of a third group with saline infusion to clarify the impact of infusion on the endometrium. Therefore, further studies are recommended with a larger sample size and examining frozen embryo transfer using G-CSF in the secretory or late follicular phase.

This study was the first to investigate the effect of G-CSF on the clinical pregnancy of women with stage-3/4 endometriosis receiving IVF. The results showed that the intrauterine injection of G-CSF exerted no significant effects on the pregnancy of women with stage-3/4 endometriosis undergoing IVF. However, studies with a larger sample size are needed to examine the routine use of G-CSF for women with endometriosis.

##  Conflict of Interest

The authors do not report any potential conflict of interest.
